# Perspectives on National Institutes of Health Funding Requirements for Racial and Ethnic Diversity Among Medical Scientist Training Program Leadership

**DOI:** 10.1001/jamanetworkopen.2023.10795

**Published:** 2023-05-01

**Authors:** Adeola Ayedun, Victoria Agbelese, Leslie Curry, Ruth Gotian, Laura Castillo-Page, Marney White, Adwoa Difie Antwi, Morgan Buchanan, Meron Girma, Danielle Kline, Chukwudum Okeke, Akshaya Raghu, Hamza Saleh, Anna Schwartz, Dowin Boatright

**Affiliations:** 1 Global Health Leadership Initiative, Yale School of Public Health, New Haven, Connecticut; 2 Department of Emergency Medicine, Yale School of Medicine, New Haven, Connecticut; 3 Department of Health Policy and Management, Yale School of Management, New Haven, Connecticut; 4 Department of Anesthesiology, Weill Cornell Medical College, New York, New York; 5 The National Academies of Sciences, Engineering, and Medicine, Washington, DC; 6 Department of Social and Behavioral Sciences, Yale School of Public Health, New Haven, Connecticut; 7 Yale School of Public Health, New Haven, Connecticut; 8 Department of Emergency Medicine, New York University Grossman School of Medicine, New York

## Abstract

**Importance:**

Since 1964, the National Institutes of Health (NIH) has funded the Medical Scientist Training Program (MSTP) MD-PhD program at medical schools across the US to support training physician-scientists. Recent studies have suggested that MSTPs have consistently matriculated more students from racial and ethnic backgrounds historically underrepresented in science than MD-PhD programs without NIH funding; however, the underlying basis for the increased diversity seen in NIH-funded MSTPs is poorly understood.

**Objective:**

To investigate how administrators and faculty perceive the impact of MSTP status on MD-PhD program matriculant racial and ethnic diversity.

**Design, Setting, and Participants:**

This qualitative study used a positive deviance approach to identify 9 high-performing and 3 low-performing MSTPs based on the percentage of students underrepresented in science who matriculated into the program between 2014 and 2018. This study, a subanalysis of a larger study to understand recruitment of students underrepresented in science at MSTPs, focused on in-depth qualitative interviews, conducted from October 26, 2020, to August 31, 2022, of 69 members of MSTP leadership, including program directors, associate and assistant program directors, and program administrators.

**Main Outcomes and Measures:**

The association of NIH funding with institutional priorities, programs, and practices related to MD-PhD program matriculant racial and ethnic diversity.

**Results:**

The study included 69 participants (mean [SD] age, 53 [10] years; 38 women [55%]; 13 African American or Black participants [19%], 6 Asian participants [9%], 12 Hispanic participants [17%], and 36 non-Hispanic White participants [52%]). A total of 51 participants (74%) were in administrative roles, and 18 (26%) were faculty involved in recruitment. Five themes emerged from the data: (1) by tying MSTP funding to diversity efforts, the NIH created a sense of urgency among MSTP leadership to bolster matriculant diversity; (2) MD-PhD program leadership leveraged the changes to MSTP grant review to secure new institutional investments to promote recruitment of students underrepresented in science; (3) MSTPs increasingly adopted holistic review to evaluate applicants to meet NIH funding requirements; (4) MSTP leadership began to systematically assess the effectiveness of their diversity initiatives and proactively identify opportunities to enhance matriculant diversity; and (5) although all MSTPs were required to respond to NIH criteria, changes made by low-performing programs generally lacked the robustness demonstrated by high-performing programs.

**Conclusions and Relevance:**

This study suggests that NIH funding requirements may be a powerful incentive to promote diversity and positively affect representation of students underrepresented in science in the biomedical scientific workforce.

## Key Points

**Question:**

How do administrators and faculty perceive the impact of National Institutes of Health (NIH) funding requirements for Medical Scientist Training Programs (MSTPs) on the racial and ethnic diversity of matriculants?

**Findings:**

In this qualitative study including 69 administrators and faculty at 12 MSTPs, NIH funding requirements for diversity fostered urgency among MSTP leadership to bolster matriculant diversity, enabled program administrators to secure additional investments in diversity, promoted holistic review, and encouraged rigorous evaluation of the effectiveness of recruitment efforts.

**Meaning:**

Findings suggest that funding requirements, such as those instituted by the NIH, may encourage impactful institutional practices to increase recruitment efforts and improve the representation of diverse candidates in training programs and, ultimately, the biomedical science workforce.

## Teaser

This qualitative study investigates how administrators and faculty perceive the impact of MSTP status on MD-PhD program matriculant racial and ethnic diversity.

## Introduction

Physician-scientists with an MD-PhD degree serve a vital role in advancing knowledge in the biomedical workforce. Although they comprise only 4% of medical school graduates, these physician-scientists receive nearly 50% of National Institutes of Health (NIH) research funding grants awarded to physicians.^1^ However, the emerging MD-PhD workforce faces many challenges, and the NIH and the US Congress have cited the lack of racial and ethnic diversity in the biomedical workforce as a key concern.^1,2^

The benefits of diversity within research teams are well documented and include a greater ability to solve complex problems,^3^ generate higher-quality research,^4,5^ and increase participation in clinical trials by underrepresented populations.^6^ Despite these benefits, in 2020, only 2% of NIH Research Project Grants (R01 grants) were awarded to Black scientists, and only 5% were awarded to Latinx scientists.^3^

Since 1964, the NIH has funded the Medical Scientist Training Program (MSTP) at medical schools across the US to support the training of physician-scientists obtaining an MD-PhD.^7^ Prior studies have reported that MD-PhD programs with MSTP status have consistently matriculated a higher percentage of students historically underrepresented in science (URiS) by race and ethnicity^8,9,10^ than MD-PhD programs without MSTP status. Notably, URiS students are nearly 4 times more likely to matriculate at longstanding MSTPs than non-MSTPs.^8^ Nevertheless, the mechanism for this higher level of diversity is poorly understood.

To address this knowledge gap, we conducted in-depth interviews with MSTP leadership among a diverse cohort of MD-PhD programs nationally to better understand URiS student recruitment practices and how MSTP status was associated with matriculant diversity with regard to race and ethnicity.

## Methods

### Study Design and Sample

This analysis is a component of a larger parent study that primarily sought to understand URiS student recruitment practices at MD-PhD programs nationally. The parent study used a positive deviance approach, comparing programs with differing levels of URiS student matriculation to generate hypotheses about factors that distinguished them.^11,12^ In deviant case sampling,^12^ the aim is to focus on examples “rich in information” that are unique in some way, which allows us to learn lessons that can be applied to other settings. We selected programs based on the percentage of URiS students^13^ who matriculated into the program between 2014 and 2018, using data from the Association of American Medical Colleges (Box 1).^9^ Because we were interested in the association of MSTP funding with matriculant diversity, we excluded non-MSTPs from the parent study. High-performing programs were defined as those in the top quartile by mean percentage of URiS student matriculants (12 sites, ≥18%), while low-performing programs were drawn from the bottom quartile (12 sites, ≤11%) (Table 1). We enrolled programs until theoretical saturation was achieved (no new concepts emerged with successive interviews).^14^ As is common in positive deviance studies,^15,16,17,18,19^ saturation required more interviews with participants from high-performing programs than low-performing programs because participants from high-performing programs had deeper experience with the phenomenon of interest. Participants provided verbal informed consent to participate, and the study was approved by an institutional review board at Yale University. This qualitative study followed the Standards for Reporting Qualitative Research (SRQR) reporting guideline.^20^

Box 1. **Positive Deviance Study Design**

**Aim 1: quantitative study to identify positive deviants**

American Medical College Application Service data provided by the Association of American Medical Colleges for academic years 2014-2018
Mean percentage of URiS matriculants per class
Stratification into quartiles; top quartile (n = 12) identified as high-performing programs and bottom quartile (n = 12) as low-performing programs
Final sample size: 9 high-performing programs and 3 low-performing programs

**Aim 2: qualitative data collection and analysis to generate hypotheses**

In-depth interviews with 162 participants at identified high- and low-performing programs
Overall thematic analysis to identify URiS student recruitment best practices and generate hypotheses; this includes a subanalysis of the whole data set, using 69 program leaders to understand the effect of NIH requirements for funding as associated with diversity

**Aim 3: quantitative study to test hypotheses through survey instrument**

Qualtrics survey with items generated from in-depth interviews
Targeted 162 respondents
Test the extent to which recruitment strategies identified are implemented

Abbreviations: NIH, National Institutes of Health; URiS, underrepresented in science.

**Table 1.** MD-PhD Program Characteristics and Interview Distribution

Site category and geographic location	No. of interviews (N = 69)
High-performing program (mean % URiS students, ≥18)	
South	11
Northeast	4
Midwest	6
South	4
Northeast	9
South	8
West	7
Midwest	5
Northeast	7
Low-performing program (mean % URiS students, ≤11)	
South	2
West	2
Northeast	4

Abbreviation: URiS, underrepresented in science.

### Recruitment

Directors of MD-PhD programs at selected institutions were contacted by the principal investigator (D.B.) via email to participate in the parent study. Of the 13 institutions that we invited to participate, 12 responded (1 institution did not reply); 9 institutions were high performing, and 3 were low performing. After providing informed consent, program directors worked with the study team to provide an initial set of potential key informants in administrative, faculty, and student roles. Key informants included program directors, chief diversity officers, deans of diversity, deans of admissions, deans of students, MD-PhD faculty involved in recruitment, faculty of racial and ethnic minority groups, and URiS MD-PhD students. Once connected with key informants, we further recruited participants via “snowball” sampling^21,22^ within the program to identify others with knowledge of efforts to increase matriculant diversity. In the parent study, we interviewed 162 individuals with diverse roles. For the present study, we focused on a subset of 69 interviews with administrators and faculty involved in recruitment because they were most likely to be knowledgeable of efforts to increase URiS student recruitment.

### Data Collection

From October 26, 2020, to August 31, 2022, we conducted in-depth individual interviews with MSTP leadership via Zoom. Our multidisciplinary team had expertise in public health, emergency medicine, psychology, and journalism; all had experience conducting in-depth interviews. Interviews typically lasted 1 hour, followed a structured interview guide (eTable 1 in Supplement 1), and were audiotaped and professionally transcribed. During data collection, participants provided their race and ethnicity, self-identifying with their preferred racial and ethnic categories, because we were interested in documenting the experiences of URiS participants in the parent study.

### Statistical Analysis

We analyzed interview data using the constant comparison method,^21,23^ coding iteratively with data collection.^12^ First, we developed a “start list”^21^ of codes based on extant literature. A team of 11 analysts (A.A., V.A., A.D.A., M.B., M.G., D.K., C.O., A.R., H.S., A.S., and D.B.), working in alternating teams of 2, coded transcripts independently and met frequently to discuss new ideas, refine the code structure, and reach consensus on divergent views. We refined the code structure until no new concepts emerged, finalizing after 15 rounds of revision (eTable 2 in Supplement 1).^24^ We used Atlas.ti Scientific Software, version 9.1 (Atlas.ti). We used standard techniques to ensure that data collection and analysis were systematic and verifiable, including discussion guides for all interviews, audiotaping and independent transcription, standardized coding and analytic procedures, multiple diverse coders, and an audit trail to document analytic decisions.^25^

## Results

The study included 69 participants (mean [SD] age, 53 [10] years; 38 women [55%]; 13 African American or Black participants [19%], 6 Asian participants [9%], 12 Hispanic participants [17%], and 36 non-Hispanic White participants [52%]). Participants represented individuals most involved in implementing changes to URiS student recruitment practices at their respective MSTPs. Informants consisted of administrators (primary roles as program directors, diversity officers, deans, coordinators, and other program staff) and faculty involved in recruitment. A total of 51 participants (74%) were administrators, and 18 participants (26%) were faculty involved in recruitment (Table 2).

**Table 2.** Participant Characteristics

Characteristic	Participants, No. (%) (N = 69)
Male	31 (45)
Female	38 (55)
Nonbinary or other	0
Race and ethnicity	
African American or Black alone	13 (19)
Asian alone	6 (9)
Hispanic	12 (17)
Native American or Alaska Native alone	1 (1)
Native Hawaiian and other Pacific Islander alone	0
White alone, non-Hispanic	36 (52)
≥2 Races	1 (1)
Role	
Administrators	
Program director	11 (16)
Associate, assistant, or deputy program director; co-program director	14 (20)
Program coordinators, managers, evaluators, other administrative directors	12 (17)
Dean (eg, admissions, student affairs, and faculty)	8 (12)
Diversity officers (eg, chief diversity officers, DEI deans, and office of multicultural affairs)	6 (9)
Faculty involved in recruitment	18 (26)

Abbreviation: DEI, diversity, equity, and inclusion.

During analysis, NIH funding requirements arose as a factor associated with URiS student recruitment activity. Respondents reflected on how changes in the way the NIH evaluated program diversity during grant reviews were associated with programmatic decisions and priorities (in 2002, in addition to preexisting criteria, the NIH revised the funding opportunity review criteria for MD-PhD programs applying for MSTP funding to include an evaluation of the program’s success in recruiting and retaining URiS students as part of the grant application and priority scoring).^10,26,27^ Five major themes (Box 2) emerged from participant experiences: (1) by tying MSTP funding to diversity, the NIH created a sense of urgency among MSTP leadership to bolster matriculant diversity; (2) MD-PhD program leadership leveraged the changes to MSTP grant review to secure new institutional investments to promote URiS student recruitment; (3) MSTPs increasingly adopted holistic review to evaluate applicants to meet NIH funding requirements; (4) MSTP leadership began to systematically assess the effectiveness of their diversity initiatives and proactively identify opportunities to enhance matriculant diversity; and (5) although all MSTPs were required to respond to NIH criteria, changes made by low-performing programs generally lacked the robustness demonstrated by high-performing programs.

Box 2. **Themes with Illustrative Quotations**

**Theme: tying MSTP funding to diversity efforts created sense of urgency among MSTP leadership to bolster matriculant diversity**

**Illustrative quotations**

We were actually docked a lot of spots because they [NIH] didn’t feel that we had done a good enough job in recruiting underrepresented minorities into the MD-PhD program. That was a very specific criticism of our application, and we had to make sure we had some way to address that…. So that was largely the impetus to try something…. When I took it over, I think I had to be a lot more proactive in trying to make it work (site 10, participant 3).
Before I started working here NIH came at [the program] and said, “You have to do better with your URM recruitment in the context of the MD-PhD program.” I think at that point is when recruitment in that particular area really started being taken care of and more attention paid and intensified (site 11, participant 8).

**Theme: program leadership leveraged changes to MSTP review to secure new investments to promote URiS recruitment**

**Illustrative quotations**

The dean was there in the room when we were being site-visited by NIH…. And so that was part of the reason that I could then kind of go to the dean, and say, “Look. It’s really important for you to give money and support these things like the undergraduate research program because NIH is paying attention, and they don’t think [the program] is doing a good job…. So, in a way, the emphasis at NIH was very helpful” (site 1, participant 1).
One of our MSTP NIH reviews wanted us to be more innovative in terms of bringing more underrepresented [students] in. So we were able to show them that and get their commitment to support the program…. We had to recently re-request funding where we showed them our data that it’s been a very successful program…. So I think some of the impetus was probably poked by the comments from the NIH grant (site 10, participant 3).
We got a summary statement back on our [year] renewal, that said, “It’s wonderful that the institution is doing these things to promote diversity and equity in admissions. But what is the MD-PhD program doing?” In response…we turned around and saw that our [dean] was going to historically Black colleges on a regular basis. He was in charge of an outreach program to [a Hispanic-serving institution]. We made him an associate director of our program as well and included him in all of the decision-making regarding the program, including him in all of the administrative meetings, the interviews, everything, so that the outreach he was doing on the part of the graduate school also became outreach on behalf of the program (site 12, participant 4).

**Theme: increased adoption of holistic review to evaluate applicants**

**Illustrative quotations**

How do you evaluate a student that…may have come a long distance—may have been the first person in the family to go to college, let alone…medical school…. And how do you balance that in the admissions process?…I think it’s getting better. I think everywhere we’re going to much more holistic admissions—getting away from using standardized tests as a measurement…. Looking at the distance traveled, is this somebody that really is interested in coming here and moving the needle and commit to the career?…I do think that the admissions process has gotten more holistic and probably more about the entire arc of the applicant, which has helped (site 8, participant 7).
When I took over the program, we had about 10% underrepresented students in the program, just like every other MSTP. And the first couple of years I was director, I focused on MCAT [scores] and GPA, and we weren’t particularly [successful] in changing the numbers. And I realized that if we don’t consider other factors and do more holistic review of the applicants than just the numbers, we’re never going to change the needle (site 9, participant 1).
The other thing that we’ve done is in recognition of the fact that there is historical racism in the American educational system that starts in kindergarten and is very hard to undo by the time you’re in college…we don’t really put as much weight on the MCAT [scores] as we used to. We used to have admissions committee meetings where someone would say, “I really like this student, but.” And the but was always a mention of a red flag somewhere in the scores on the MCAT. And now we don’t discuss the MCAT [scores] after we meet the students in person. If we bring them in for in-person interviews, it’s understood that all of those numeric scores, your past, you’ve moved beyond those now, and now we’re looking for other qualities in the applicants that would make them good members of our MD-PhD community…. We are trying to make sure that we don’t sort of miss those diamonds in the rough (site 11, participant 17).

**Theme: systematic assessment of effectiveness of diversity initiatives and proactive identification of opportunities to enhance diversity**

**Illustrative quotations**

The number of underrepresented minority medical students has gone through the roof. When I became the director, it was about a third women and two-thirds men. Now it’s just about 50-50. I doubled the number of underrepresented minority students during my time from about 10 to 20-something percent. And we were patted on the back, lauded by the NIH for having such a high percentage of underrepresented minority students for such a selective program…. Traditionally it’s been much easier for us to recruit Latino, Latinx underrepresented minority students, [but not] African American students…. But we’ve been chipping away at that and doing better with it (site 8 participant 1).

**Theme: changes made by low-performing programs generally lacked robustness of high-performing programs**

**Illustrative quotations**

We know we are receptive to some of the things that NIH needs. Of course, we’re not just doing it for that, but we have to be aware of that because then otherwise we would lose our own funding. So it’s a balance of who we want to attract and have in our program and mentor them, plus who do we need to answer to make sure that our program is considered top notch and sustained long term (site 4, participant 2).
I think our success in recruiting individuals from diverse and marginalized groups has not been as good as I have hoped. We’re sort of holding our own. But we’re not making huge strides in that…. I think we’ve got a long way to go. I think we’ve got a lot more work (site 5, participant 4).
It is something at the forefront of our mind. I mean, we want to make sure that we are meeting NIH benchmarks and our reporting is accurate…. It’s something we’re aware of. If we have a year where we don’t have any underrepresented minorities, it’s something that is obvious, something that we talk about, maybe why they didn’t accept our offer, what we can do next year to successfully recruit those applicants (site 6, participant 85).

Abbreviations: GPA, grade point average; MCAT, Medical College Admission Test; MSTP, Medical Scientist Training Program; NIH, National Institutes of Health; URiS, underrepresented in science; URM, underrepresented in medicine.

### First Theme

By tying MSTP funding to diversity efforts, the NIH created a sense of urgency among MSTP leadership to bolster matriculant diversity. Members of MSTP leadership reported that the NIH’s linking of MSTP funding to diversity efforts created an immediate impetus to prioritize the recruitment of URiS students.

We have to provide a diversity report of recruitment with our application…it was not a very good report for the previous 5 years…a couple of years there were no students that fit the diversity category. The grant went in…about a month later, we got this letter that said, “We’re disallowing the award because we think your diversity record and your plans for diversity are inadequate for our funding.” So therein began a number of changes in the program (site 1, participant 6).

Shifts in NIH funding evaluation criteria realigned program priorities to focus on increasing diversity. As 1 assistant program director stated,

NIH is asking lots of tough questions about diversity. When we go to renew our training grant, they’re asking for all these numbers and asking us what do we do to try to increase diversity in our program (site 10, participant 1).

New diversity funding criteria forced MD-PhD program leadership to thoroughly reflect on and document their prior work to support diversity, which created a mechanism of accountability that did not previously exist. One MSTP administrator noted,

I had to write this report to NIH about what we had done with minority students…. That was probably the most important thing because I realized that we had not done as much as I thought we should have done… (site 7, participant 1).

Some MD-PhD program leadership shared receiving negative feedback from the NIH regarding low matriculant diversity on MSTP grant applications. In a few cases, MD-PhD programs were given probationary standing because of a consistent lack of diversity.

Many of the questions that they [NIH reviewers] had were about our ability to recruit minority students to our program, to retain them in training, and to have successful outcomes… We only had probationary funding, and it was contingent upon our improving our record (site 1, participant 1).

The risk of losing funding spurred institutional activity to enhance URiS student recruitment. Respondents at programs placed on probation noted a change in their program’s willingness to pursue diversity initiatives after their NIH funding was jeopardized. One program administrator described being “threatened with having the program not funded anymore” as an event that “crystallizes one’s thoughts and actions” (site 1, participant 6). For example, some institutions describe implementing action plans to support increased enrollment of URiS candidates.

When I became director…I realized that NIH actually had put us on probation for our lackluster performance with respect to minority students…when I took over, it was very traumatic… I laid out an action plan for how we could overcome these problems (site 7, participant 1).

### Second Theme

Leadership of MD-PhD programs leveraged the changes to MSTP grant review to secure new institutional investments to promote URiS student recruitment. Pressure from the NIH to improve program diversity promoted buy-in for recruitment strategies among administrators and yielded financial investments in human capital and program activities to support diversity. One administrator described the confluence of the program director’s goal of improving diversity with the provision of resources to hire an individual to support those efforts.

When we had one of our reviews for our NIH grant there was a comment that we needed to increase the diversity of the program. Then that was the goal of the directors as well. So, between the NIH, “You need to up the game a little bit,” and the interest on the directors’ part, it became a more formalized process when they hired the [URiS recruiter] (site 11, participant 11).

At the institution described, the administration allocated money for an individual to attend conferences geared toward supporting URiS students, such as the Annual Biomedical Research Conference for Minoritized Scientists (ABRCMS). In addition, this individual received financial resources to start college tours, which included visiting smaller schools and historically Black colleges and universities (HBCUs) to recruit students. At other institutions, administrators were able to lobby for increased funds to establish institutional infrastructure that could help increase and develop the URiS candidate pool, such as summer research programs.

### Third Theme

Medical Scientist Training Programs increasingly adopted a holistic review to evaluate applicants to meet NIH funding requirements. To increase the number of URiS students, programs altered their approach and developed strategies to evaluate candidates holistically. With a holistic review of candidates, programs evaluate applicants’ capabilities in a way that allows for a balanced consideration of their experiences, academic performance, and characteristics that may contribute positively to their success as students and physician-scientists.^28^

One of the official complaints of the NIH reviews of our program…was that we didn’t have a very diverse student body. [Program director] came on board and really moved the admission process and considerations of the applications to a much more holistic review that included URM [underrepresented minority] status, disadvantage status, and this distance traveled criterion (site 1, participant 8).

Programs made efforts to create applicant review teams that were also more diverse and reflected the makeup of candidates that they aimed to recruit. In addition, programs reported the responsiveness to changes in the NIH’s definition of diversity^27,29^ and what populations are considered URiS, resulting in expansion of the demographic characteristics that they target for recruitment.

The NIH expanded the definition to include now a low socioeconomic status… One of the things that I did…when I became program director is I changed the formula that we use for evaluating applications (site 3, participant 2).

### Fourth Theme

Medical Scientist Training Program leadership began to systematically assess the effectiveness of their diversity initiatives and proactively identify opportunities to enhance matriculant diversity. As they complied with NIH standards, many programs described initiating rigorous assessments of the effectiveness of their recruitment strategies. As 1 program director identified, they noted disparities in sex and among specific racial and ethnic URiS groups, some of which they had improved, and others they knew had yet to be fully addressed.

We have this database so we can look and see how are we doing…we have a column to identify, are we talking to underrepresented students in this group? What’s the percentage in this group? Is the institution Hispanic-serving or Hispanic Association of Colleges and Universities or HBCU? …We have those identifiers…track what are you doing…then you can find areas to grow…we do that in a purposeful way (site 3, participant 14).

Furthermore, as NIH diversity reporting criteria have evolved,^30^ so have the ways in which institutions track the makeup of their classes. For example, 1 assistant program director stated,

NIH requires a trainee diversity report with every annual progress report that we provide…those numbers have to be tracked in order for the NIH partner support to be completed. The last 2 years have been really going back to rigorous data standards to be able to track those demographic data. Because they weren’t rigorously tracked over time (site 1, participant 8).

### Fifth Theme

Although all MSTPs were required to respond to NIH criteria, changes made by low-performing programs generally lacked the robustness demonstrated by high-performing programs. Some low-performing institutions differed from high-performing MSTPs in the intensity of their activity to recruit URiS students. Administrators at low-performing institutions described being “receptive” to NIH requirements and being “aware” otherwise they risk losing their funding (site 4, participant 2).

However, while the NIH standards promoted an understanding of a need to increase diversity and encouraged recruitment activity, some administrators at these MD-PhD programs acknowledged the lack of an institutional “why” (eg, purpose, commitment, mission) as a hindrance to progress. Efforts at low-performing programs seemed to be more perfunctory without complete buy-in to promote diversity.

Historically, we’ve gone to these recruitment fairs because we are supposed to, because as an NIH-funded grant program, we’re supposed to. But it’s really kind of separating what we should be doing vs why do we want to do this? I think that’s kind of been a challenge. All these policies were in place just because we had to have policies. But I think the institution and our program is really realizing that you need to have the “why” within you, or else, it’s not going to work… I think that’s been a challenge, getting the genuineness to move us forward (site 5, participant 1).

Although some low-performing programs struggled with establishing a strong commitment to improving URiS student recruitment activities, this was not the case for all, with some noting it as important regardless of NIH funding requirements. At these programs, administrators see URiS student recruitment as “something [they] put a lot of effort into” and “don’t necessarily need a financial incentive for” but acknowledged being “pragmatic… to keep NIH funding” (site 6, participant 84).

Overall, while low-performing programs responded to NIH funding requirements, they were not as proactive as high-performing institutions where administrators realized they had to “stay ahead of the curve” (site 11, participant 17). These institutions were more likely to rely on fewer activities, such as attendance at ABRCMS, and had only more recently started collaborations that would allow them to recruit students through initiatives such as pipeline programs. This differed from high-performing institutions that had more established initiatives, which potentially demonstrate increased investment of human and financial resources.

We’re trying to start a pipeline program. We’re hoping over time, maybe we can open it up broader than [our school] and attract students from other campuses (site 4, participant 3).

Finally, some low-performing programs differed from high-performing programs in their reliance on traditional application metrics, such as standardized test scores, for admission as opposed to more holistic reviews of candidates. Some described these metrics as a challenge to their recruitment efforts.

## Discussion

We found that changes to MSTP grant review linking funding to diversity compelled programs to adopt efforts to increase matriculant diversity. Efforts included redefined programmatic priorities geared toward improving diversity, the allocation of financial and human resources to bolster matriculant diversity, the use of holistic review to enrich the applicant pool, and assessment to determine effectiveness of recruitment strategies.

These findings are consistent with studies that demonstrate an association between medical school accreditation standards and subsequent gains in matriculant diversity.^31^ Although MSTPs have consistently achieved higher rates of racial and ethnic diversity compared with non-MSTPs,^9,32^ this study is the first, to our knowledge, to describe the ways in which NIH funding requirements have been associated with MD-PhD program efforts to improve diversity. This includes required detailed documentation of diversity efforts and outcomes as part of MSTP grant funding review.^33^

The study findings have implications for national funding agencies and MD-PhD programs. National Institutes of Health–funded MSTPs account for approximately 44% of all MD-PhD programs in the US.^34,35^ Given the potential of MSTPs to successfully recruit URiS matriculants, expanding the number of programs could present a significant opportunity to increase diversity among physician-scientists. Furthermore, increasing the number of training slots at current MSTPs may be another strategy to increase MD-PhD program diversity.

Behavioral and economic incentives to promote diversity within MSTPs created by NIH funding criteria, if applied to other NIH grant types, could lead to increased diversity in the entire biomedical scientific research enterprise. Medical schools receive nearly 67% of all NIH funding.^36^ Given this substantial financial support, the NIH could extend its influence to promote diversity beyond MSTP funding to all grant awards, creating a strong impetus for academic medical centers to broadly bolster diversity.

The NIH could link medical school diversity efforts to federal funding by several means. The research environment is a key component of evaluation for most NIH grants. The NIH could require grant applicants to report institutional measures of diversity, equity, and inclusion, such as faculty compositional diversity, promotion and attrition rates by race and ethnicity, and pay equity, which could be assessed as part of the research environment in grant priority scoring. Similarly, the NIH could require grant applicants to describe efforts to assemble a team of investigators from diverse backgrounds, especially given prior literature demonstrating that diverse teams may produce higher-quality science.^3,4,5^ These efforts could be evaluated in the investigators section of NIH grant priority scoring.

Although the NIH’s linking of diversity to MSTP funding may have a beneficial, forcing-function influence on programs and may encourage diversity recruitment activities, study findings suggest that this policy may not be sufficient to fully mitigate the disparities that we see in the biomedical research workforce. Some programs may adhere to the criteria with perfunctory or “checking the box” behavior that may not lead to sustainable parity in representation. The diversity policy alone may also be insufficient to promote a sincere belief in the importance of diversity in science and may result in programs meeting minimum standards only to maintain NIH funding. Beyond the findings of this study, other considerations exist that may affect the impact of the NIH’s diversity policy. For example, future studies should examine how judicial rulings related to affirmative action anticipated at the Supreme Court may affect the NIH’s ability to promote diversity in the physician-scientist workforce.

### Limitations

This study has some limitations, and these data must be interpreted in the context of the study design. First, our criteria for selecting MSTPs that are high and low performing, as determined by the number of URiS matriculants, may be associated with other factors beyond the programs’ control, such as size of the institution, geographic location, and perceived institutional reputation. Second, our sample is limited to NIH-funded MSTP MD-PhD programs. Although NIH-funded MSTPs comprise two-thirds of all MD-PhD students, our study does not explore how NIH funding opportunity review criteria may have been indirectly associated with programs without NIH support. Third, we acknowledge that there may be recall bias and social desirability that may have affected participants’ responses.^37^ To mitigate these factors, we included interview probes designed to elicit both positive and negative experiences from participants,^38^ provided assurances of confidentiality, and interviewed a wide range of individuals from all levels of the institutions to ensure we had diverse perspectives.

## Conclusions

In our qualitative study of MSTPs, we found that the NIH’s linking of diversity efforts to MSTP funding created a powerful impetus for program leadership to enhance the recruitment and retention of URiS students. The NIH could consider linking diversity efforts to additional funding opportunities to increase the diversity of the biomedical research enterprise.

## Notes

Supplement 1. **eTable 1.** Interview Guide **eTable 2.** Final Code Structure, Version 15

Supplement 2.
Data Sharing Statement

## References

[1] Physician-Scientist Workforce Working Group report. National Institutes of Health. 2014. Accessed October 27, 2022. https://acd.od.nih.gov/documents/reports/PSW_Report_ACD_06042014.pdf 10.1111/cts.12209 PMC5439807 25123835

[2] National Academy of Sciences, National Academy of Engineering, and Institute of Medicine. Expanding Underrepresented Minority Participation: America’s Science and Technology Talent at the Crossroads. National Academies Press; 2011:286.

[3] Hong L, Page SE. Groups of diverse problem solvers can outperform groups of high-ability problem solvers. Proc Natl Acad Sci U S A. 2004;101(46):16385-16389. doi:10.1073/pnas.0403723101 15534225 PMC528939

[4] Campbell LG, Mehtani S, Dozier ME, Rinehart J. Gender-heterogeneous working groups produce higher quality science. PLoS One. 2013;8(10):e79147. doi:10.1371/journal.pone.0079147 24205372 PMC3813606

[5] Freeman RB, Huang W. Collaborating with people like me: ethnic coauthorship within the United States. J Labor Econ. 2015;33(S1):S289-S318. doi:10.1086/678973

[6] Noah BA. The participation of underrepresented minorities in clinical research. Am J Law Med. 2003;29(2-3):221-245. doi:10.1017/S0098858800002823 12961806

[7] Harding CV, Akabas MH, Andersen OS. History and outcomes of 50 years of physician-scientist training in medical scientist training programs. Acad Med. 2017;92(10):1390-1398. doi:10.1097/ACM.0000000000001779 28658019 PMC5617793

[8] Jeffe DB, Andriole DA. A national cohort study of MD-PhD graduates of medical schools with and without funding from the National Institute of General Medical Sciences’ Medical Scientist Training Program. Acad Med. 2011;86(8):953-961. doi:10.1097/ACM.0b013e31822225c5 21694566 PMC3145809

[9] Martinez-Strengel A, Samuels EA, Cross J, . Trends in U.S. MD-PhD program matriculant diversity by sex and race/ethnicity. Acad Med. 2022;97(9):1346-1350. doi:10.1097/ACM.0000000000004747 35583935 PMC9474393

[10] Investing in the future: strategic plan for biomedical and behavioral research training. National Institute of General Medical Sciences. 2011. Accessed October 27, 2022. https://www.nigms.nih.gov/about/Documents/NIGMS-Strategic-Training-Plan.pdf

[11] Marsh DR, Schroeder DG, Dearden KA, Sternin J, Sternin M. The power of positive deviance. BMJ. 2004;329(7475):1177-1179. doi:10.1136/bmj.329.7475.1177 15539680 PMC527707

[12] Patton MQ. *Qualitative Research & Evaluation Methods*. 3rd ed. Sage Publications; 2002.

[13] Nguyen M, Chaudhry SI, Desai MM, . Rates of medical student placement into graduate medical education by sex, race and ethnicity, and socioeconomic status, 2018-2021. JAMA Netw Open. 2022;5(8):e2229243. doi:10.1001/jamanetworkopen.2022.29243 36018592 PMC9419015

[14] Morse JM. The significance of saturation. Qual Health Res. 1995;5(2):147-149. doi:10.1177/104973239500500201

[15] Bradley EH, Curry LA, Spatz ES, . Hospital strategies for reducing risk-standardized mortality rates in acute myocardial infarction. Ann Intern Med. 2012;156(9):618-626. doi:10.7326/0003-4819-156-9-201205010-00003 22547471 PMC3386642

[16] Brewster AL, Brault MA, Tan AX, Curry LA, Bradley EH. Patterns of collaboration among health care and social services providers in communities with lower health care utilization and costs. Health Serv Res. 2018;53(suppl 1):2892-2909. doi:10.1111/1475-6773.12775 28925041 PMC6056597

[17] Brewster AL, Curry LA, Cherlin EJ, Talbert-Slagle K, Horwitz LI, Bradley EH. Integrating new practices: a qualitative study of how hospital innovations become routine. Implement Sci. 2015;10:168. doi:10.1186/s13012-015-0357-3 26638147 PMC4670523

[18] Curry L, Cherlin E, Ayedun A, . How do area agencies on aging build partnerships with health care organizations? Gerontologist. 2022;62(10):1409-1419. doi:10.1093/geront/gnac019 35092437

[19] Curry LA, Spatz E, Cherlin E, . What distinguishes top-performing hospitals in acute myocardial infarction mortality rates? a qualitative study. Ann Intern Med. 2011;154(6):384-390. doi:10.7326/0003-4819-154-6-201103150-00003 21403074 PMC4735872

[20] O’Brien BC, Harris IB, Beckman TJ, Reed DA, Cook DA. Standards for reporting qualitative research: a synthesis of recommendations. Acad Med. 2014;89(9):1245-1251. doi:10.1097/ACM.0000000000000388 24979285

[21] Miles MB, Huberman AM. *Qualitative Data Analysis: An Expanded Sourcebook*. 2nd ed. Sage Publications; 1994:xiv, 338.

[22] Kuzel A. Sampling in qualitative inquiry. In: Crabtree BF, Miller WL, eds. Doing Qualitative Research. 2nd ed. Sage Publications; 1999.

[23] Glaser BG, Strauss AL. The Discovery of Grounded Theory: Strategies for Qualitative Research. Aldine Publishing Co; 1967.

[24] Deterding NM, Waters MC. Flexible coding of in-depth interviews: a twenty-first–century approach. Sociol Methods Res. 2021;50(2):708-739. doi:10.1177/0049124118799377

[25] Curry LA, Nembhard IM, Bradley EH. Qualitative and mixed methods provide unique contributions to outcomes research. Circulation. 2009;119(10):1442-1452. doi:10.1161/CIRCULATIONAHA.107.742775 19289649

[26] NIH National Research Service Award institutional research training grants (T32). National Institutes of Health. Accessed November 10, 2022. https://grants.nih.gov/grants/guide/pa-files/PA-02-109.html

[27] Notice of NIH’s interest in diversity. National Institutes of Health. Accessed October 27, 2022. https://grants.nih.gov/grants/guide/notice-files/NOT-OD-20-031.html

[28] Holistic review in medical school admissions: an introduction. Association of American Medical Colleges. Accessed February 23, 2023. https://www.aamc.org/media/45551/download

[29] Updated notice of NIH’s interest in diversity. National Institutes of Health. Accessed February 23, 2023. https://grants.nih.gov/grants/guide/notice-files/NOT-OD-18-210.html

[30] Requirement for trainee diversity report to be submitted electronically for institutional research training, career development, and research education awards beginning in early FY 2021. National Institutes of Health. Accessed February 23, 2023. https://grants.nih.gov/grants/guide/notice-files/NOT-OD-20-178.html

[31] Boatright DH, Samuels EA, Cramer L, . Association between the Liaison Committee on Medical Education’s diversity standards and changes in percentage of medical student sex, race, and ethnicity. JAMA. 2018;320(21):2267-2269. doi:10.1001/jama.2018.13705 30512090 PMC6583477

[32] Jeffe DB, Andriole DA, Wathington HD, Tai RH. The emerging physician-scientist workforce: demographic, experiential, and attitudinal predictors of MD-PhD program enrollment. Acad Med. 2014;89(10):1398-1407. doi:10.1097/ACM.0000000000000400 25006709 PMC4175019

[33] Funding opportunity announcement: medical scientist training program (T32). National Institutes of Health. Accessed February 23, 2023. https://grants.nih.gov/grants/guide/pa-files/PAR-21-189.html

[34] MD-PhD degree programs by state. Association of American Medical Colleges. Accessed October 27, 2022. https://students-residents.aamc.org/applying-md/phd-programs/md-phd-degree-programs-state

[35] Medical Scientist Training Program. National Institute of General Medical Sciences. Accessed October 27, 2022. https://nigms.nih.gov/training/instpredoc/Pages/PredocOverview-MSTP.aspx#:~:text=The%20MSTP%20currently%20has%2050%20participating%20programs%20and%20supports%20approximately%201000%20trainees 10.34197/ats-scholar.2022-0018PS PMC9585701 36312807

[36] Gibbs KD, Basson J, Xierali IM, Broniatowski DA. Decoupling of the minority PhD talent pool and assistant professor hiring in medical school basic science departments in the US. eLife. 2016;5:e21393. doi:10.7554/eLife.21393 27852433 PMC5153246

[37] Bradburn NM, Sudman S, Wansink B. Asking Questions: The Definitive Guide to Questionnaire Design—For Market Research, Political Polls, and Social and Health Questionnaires. Revised ed. Jossey-Bass; 2004.

[38] Mays N, Pope C. Qualitative research in health care: assessing quality in qualitative research. BMJ. 2000;320(7226):50-52. doi:10.1136/bmj.320.7226.50 10617534 PMC1117321

