# Supplementary material for: Perspectives on National Institutes of Health Funding Requirements for Racial and Ethnic Diversity Among Medical Scientist Training Program Leadership
Source: JAMA Netw Open. 2023 May 1;6(5):e2310795. doi: 10.1001/jamanetworkopen.2023.10795 (PMC10152303; doi:10.1001/jamanetworkopen.2023.10795)
Supplement: Supplement 1. — eTable 1. Interview Guide eTable 2. Final Code Structure, Version 15 [file jamanetwopen-e2310795-s001.pdf]

Supplementary Online Content

Ayedun A, Agbelese V, Curry L, et al. Perspectives on National Institutes of Health funding requirements for racial and ethnic diversity among Medical Scientist Training Program leadership. *JAMA Netw Open.* 2023;6(5):e2310795. doi:10.1001/jamanetworkopen.2023.10795

eTable 1. Interview Guide
eTable 2. Final Code Structure, Version 15

This supplementary material has been provided by the authors to give readers additional information about their work.

© 2023 Ayedun A et al. *JAMA Network Open.*

eTable 1. Interview Guide

1. Let's start by having you describe what you do here?
   Possible probes:

   - *How long have you worked here? How long have you worked in this job?*
   - *What are your formal responsibilities?*

2. How do you recruit racial/ethnic (Black, Latinx, Native American, etc.) students that have been historically underrepresented among physician-scientists to the MD-PhD program? Can you walk me through the process?
   Possible probes:

   - *How does your institution build an applicant pool? Who is involved in the process?*
   - *Have there been efforts to increase the racial/ethnic diversity of MD-PhD students in your program? What got it started?*
   - *How does your program recognize problems or opportunities? How do you deal with setbacks?*
   - *Can you describe things that needed to be ironed out along the way?*

3. Has your process for recruiting racially/ethnically diverse candidates always worked this way? If it has changed, can you tell me about when that happened and how it went?
   Possible probes:

   - *Can you tell me how the changes came about?*
   - *What kinds of difficulties had to be worked through?*
   - *Can you describe changes you've noticed in the recruitment of MD-PhD students secondary to mandates from the NIH?*
   - *In what ways does your institution's relationship with the NIH/NIH policy impact the application selection process of your MD-PhD program?*

4. Can you describe how students from historically underrepresented backgrounds are supported in your program after matriculation?
   Possible probes:

   - *Describe any initiatives, programs, or committees that exist.*
   - *What are some of the things that have worked well? What kind of difficulties had to be worked through?*

5. Is there anything else I should have asked to help us better understand recruitment practices at your institution?
   Possible probes:

© 2023 Ayedun A et al. *JAMA Network Open.*

- Any surprises? Lessons learned?

© 2023 Ayedun A et al. *JAMA Network Open.*

eTable 2. Final Code Structure, Version 15

**100.**	**External Context** (the larger context, descriptions of ecological factors outside the institution that may affect recruitment, DEI, student/faculty/administrator experiences, and others)
100a.	National unrest (George Floyd, 2020 BLM protests, political climate, anti-Asian hate demonstrations)
100b.	Regulatory influences (LCME 2009 accreditation standards, MSTP status, NIH MSTP diversity funding policies, national/state/local legislation around recruitment by race, affirmative action legislation, MSTP status in jeopardy due to diversity)
100c.	COVID-19 (impact on institutional culture, provisions to support students, new practices in recruitment as a result of virtual reality, racial disparities in COVID)
**200.**	**Program Features** (specific aspects/features that describe the institution; can be the medical, graduate, or MD-PhD broadly; codes 200e, 200f, 200g may be double coded to specify program being described)
200a.	Funding (financial support from institution for students like microloans, multiple funding streams to support program, financial constraints)
200b.	Diversity (of faculty, students, whole program, leadership representation from URiS groups (i.e. Black, Latinx, Native American, women, etc.))
200c.	URiS retention (of faculty, students)
200d.	Communication/information sharing (interdepartmental, between MD and PhD portions of program)
200e.	MD program
200f.	PhD program
200g.	MD-PhD program
200h.	Institution ranking/perceived prestige
200i.	Location/region/demographics of the surrounding community (diversity, SES, urban vs rural)
200j.	Admissions process (autonomy of MD-PhD admissions committee, final say, burden in application review, single reviewer versus democratic process, formal scoring of applications)
200k.	Size of institution (as it relates to having more diverse community)
**300.**	**Student Experience** (both before and after matriculation)
300a.	Social networks and peer support systems (minority student organizations, organizations related to diversity, finding MD or PhD community)
300b.	Application process (interview day, standardized testing, clarity/transparency from institutions about requirements)
300c.	Motivation for pursuing MD-PhD (general exposure to physician-scientists, to serve specific communities)
300d.	Knowledge of navigating systems (knowing how to transition from MD to PhD, how to apply to MD-PhD, requesting letters of recommendation, how to prepare for STEP exams)

© 2023 Ayedun A et al. *JAMA Network Open.*

300e.	Personal barriers/challenges (experiences/family responsibilities that compete with academics, financial barriers, non-traditional student (age))
300f.	Mental health experiences/supports (descriptions of mental health challenges like depression, utilizing mental health resources on or off campus, institution supporting students in navigating trauma)
300g.	Academic barriers (needing specific resources like money for textbooks, difficulties in courses, retaking exams, failing out of program)
300h.	Clinical experiences
300i.	PI/lab experiences (both at current institution and prior, relationship with PI, ability to engage in research of interest, difficulties with peers in lab)
300j.	Institutional (structural) support (pre-medical advising, transition support to PhD, academic support from tutors, faculty members, access to admin/faculty for academic/non-academic challenges)
300k.	Institutional responsiveness to student non-academic needs (providing resources for students with families)
300l.	Post undergraduate programs and degrees (post-bacc, MS/MPH/other advanced degree, certification programs to improve application competitiveness)
300m.	Undergraduate experience
300n.	Family support
300o.	Motivation for engaging in program's recruitment activities
300p.	Strength of application (preparedness, gaps in application, competitiveness, lack of research experience
300q.	Deciding factors for choosing institution (research availability, diversity of community, availability of funding)
**400.**	**Recruitment Strategies** (descriptions of specific recruitment strategies and how they were implemented)
400a.	Pipeline programs [between MD-PhD and students] (internal to institution and external, establishing research opportunities for high school and/or undergraduate students, formal and informal, long-term relationships with schools, information sharing/knowledge building, emphasis on early part of pipeline (i.e. middle school))
400b.	Internal collaborations (between admissions office and diversity offices, different departments recruiting students together)
400c.	External collaborations excluding universities (private sector, NIH, NIH post-bacc)
400d.	Student involvement (student-led recruitment and overall participation, shadowing current students, social events/dinners, students recruiting students, informal recruiting, snowball, diverse students attract other diverse students, supporting other URiS in application process)
400e.	Partnership programs [between MD-PhD and other institution] (formal, at HBCUs, high schools/local community, feeder schools, reaching out to local schools/community)
400f.	Relationship-building (making students feel invited, creating a warm climate, establishing long-term relationships, empowerment, encouraging students, familiarizing families with MD-PhD, "recruiting" students' parents)

© 2023 Ayedun A et al. *JAMA Network Open.*

400g. Admissions committee (diversity, make-up, expertise in evaluation of admissions committee, competency)
400h. Holistic review of individual student applications (looking beyond GPA, MCAT, considering extracurriculars and experience)
400i. Recruitment fairs/conferences (NIH grad fair, ABRCMS, SACNAS, AAMC minority fair, graduate fairs, pre-health fairs hosted by universities/groups of universities in a region, AMEC)
400j. Evaluation of strategies (effectiveness, performance metrics, finding evidence of structural bias, responding to students' financial and other challenges, using secondary applications to gather data on effectiveness of strategies, incorporating student feedback, data driven outreach, results of strategies - diversity of applicant pool, tracking, using data for decision-making, ensuring consistency of evaluation)
400k. Changes/accommodations to promote diversity (increased recruitment, increasing number of interviews offered, size of applicant pool, accessibility of applying, switch in application processing system like AMCAS, allowing GRE scores, waiving MCAT, application fee waivers for secondary, interview day travel funding)
400l. Selling the institution (learning environment, competitiveness with other institutions, funding availability to attract students)
400m. Demographic-specific outreach to sub-groups within URiS (targeted approaches according to race/ethnicity/SES/etc.)
400n. Institution-specific definition of diversity (expanding what counts as diversity, like religious diversity, acknowledge gaps in recruitment of URiS)
400o. Holistic review of class/program as a whole (attention to class makeup, deciding future second batch offers based on current acceptances)
400p. Pipeline problem (not having enough URiS students in pool of possible applicants to pull from, competing for same students, time commitment/investment in developing diverse student body)
400q. Online outreach (use of webinars, social media, YouTube, web-based recruiting, can include informal interactions/unofficial partnerships with institutions)
400r. Design of recruitment pre-acceptance (interview day, diversity supplement)
400s. Design of recruitment post-acceptance (second-look days)

500. **Culture/Climate** (of the institution, this includes elements as they may support DEI)
500a. Learning environment (encouraging peer-to-peer exchange, grading systems, psychological safety, where students sit in classroom, competition among students, evaluating biases in grading system)
500b. Relationships (with faculty and peers, belonging/isolation)
500c. Silos (existence of silos between MD and PhD DEI offices, breaking down separation between departments in recruitment process, between admissions and office of diversity)
500d. Transparency (between students and administration/faculty about academic performance, supportive vs. challenging PIs, feeling comfortable to voice concerns)

600. **URiS Experiences** (to be used when participants (faculty, staff, or student) speak of anything relating to URiS identity and its effect on their experience at the institution)

© 2023 Ayedun A et al. *JAMA Network Open.*

600a.	Narratives/anecdotes (of positive or negative experiences, faculty experiences at institution, regretting choice of institution)
600b.	Minority tax (URiS faculty/students engaging more in DEI/recruitment, burn out from DEI activities, diversity ambassador, burdened, institution compensating/not compensating for time and effort, pressure to stay at institution as only one/engage in DEI)
600c.	Intersectionality (describing multiple facets of identity, gender, SES, having a combination of identities unique to institution/unsupported by institution)
600d.	Imposter syndrome (self-doubt, deserving to be at institution)
600e.	Acculturative stress (adapting, only one, fatigue and/or stress of being POC)
600f.	Stigma visibility (impact of visible and concealable stigmatized identity, self-perception, others not viewing individual as URiS, assimilation, concealing parts of identity, selective revealing of identities)
600g.	Faculty/Admin (to be co-coded with any of the 600s)
600h.	Student (to be co-coded with any of the 600s)
600i.	Student advocacy (students driving institutional climate/culture changes, “bottom up advocacy vs. top down”)
600j.	Career trajectory (changing specialties because of rotation/clinical experience, expectation of retaliatory/poor recommendation letters, positive guidance from faculty and mentors)
**700.**	**DEI Strategies** (tools used by the institution to promote a positive, inclusive culture/learning environment, must be specific strategies or concrete actions)
700a.	DEI approach (restructuring mission statements, climate surveys, starting DEI committees, hiring URiS faculty, diversity officers, office of diversity, changes to DEI strategy)
700b.	Conversations around identities (including race, anti-racism workshops, trainings for faculty and staff, LGBTQ, gender, openness of institution and its members)
700c.	Culturally competent curriculum (teaching cultural competency, changing curriculum from outdated ideas on race or tone-deaf instruction)
700d.	Student activity (participation in DEI initiatives, student voice on DEI initiatives, motivation to participate/support DEI, founding student organizations)
700e.	Champions (individuals named/who can be described as advocates for DEI or changes to recruitment to improve diversity, descriptions of advocacy at the institution, personal motivation for or commitment to DEI)
700f.	Administrative support (access to administrators, “showing up for students”, gatekeeping initiatives, buy in for recruitment/DEI from administrators, faculty, perceived value, funding from diversity office for recruitment, travel to recruitment affairs, to start pipeline/other DEI-related programs)
700g.	Evaluation of DEI initiatives (student/admin perceptions of DEI Initiatives, value, no longer seeing value or effect of DEI, “performative”)
**800.**	**Facilitators** (double code with other code(s) to describe things that facilitate progress, enable success, support interventions, “smooth the way”)
**900.**	**Barriers** (double code with other code(s) to describe things that get in the way, prevent progress)

© 2023 Ayedun A et al. *JAMA Network Open.*

**1000.**	**Success** (can be double coded with others like Recruitment strategies; can include descriptions of evaluation and feedback)
**2000.**	**Failure** (can be double coded with others like Recruitment strategies; can include descriptions of evaluation and feedback)
**3000.**	**New ideas and concepts not included in codes above**
**4000.**	**Opportunities/Recommendations**
**5000.**	**Mentorship** (from faculty, student perspective)
5000a.	Mentorship within MD-PhD institution (faculty advising, peer-to-peer mentorship, faculty experience mentoring URiS)
5000b.	Mentorship outside MD-PhD program (sharing research opportunities, connections to PIs, guidance on how to apply, teaching the culture of science/academia)
**6000.**	**Discrimination** (bias, stereotypes, microaggressions)
6000a.	Racism
6000b.	Sexism
6000c.	Ageism
6000d.	Sexual orientation
6000e.	Gender identity
6000f.	Institutional responses to discriminatory/racial instances/microaggressions (public statements)
**7000.**	**Jadedness**
**9999.**	**Great Quotes**

© 2023 Ayedun A et al. *JAMA Network Open.*
