# Supplementary material for: Perspectives on National Institutes of Health Funding Requirements for Racial and Ethnic Diversity Among Medical Scientist Training Program Leadership
Source: JAMA Netw Open. 2023 May 1;6(5):e2310795. doi: 10.1001/jamanetworkopen.2023.10795 (PMC10152303; doi:10.1001/jamanetworkopen.2023.10795)
Supplement: Supplement 2. — Data Sharing Statement [file jamanetwopen-e2310795-s002.pdf]

Data Sharing Statement

Ayedun. Perspectives on National Institutes of Health Funding Requirements for Racial and Ethnic Diversity Among Medical Scientist Training Program Leadership. *JAMA Netw Open.* Published May 01, 2023. doi:10.1001/jamanetworkopen.2023.10795

Data
Data available: No
